# Breast milk DHA levels may increase after informing women: a community-based cohort study from South Dakota USA

**DOI:** 10.1186/s13006-016-0099-0

**Published:** 2017-01-28

**Authors:** Brian A. Juber, Kristina Harris Jackson, Kristopher B. Johnson, William S. Harris, Michelle L. Baack

**Affiliations:** 1 Sanford School of Medicine, University of South Dakota, 1400 W. 22nd St., Sioux Falls, SD 57105 USA; 2 OmegaQuant Analytics, LLC, 5009 W. 12th St, Ste 8, Sioux Falls, SD 57106 USA; 3 Sanford Research, Children’s Health Research Center, 2301 E. 60th Street North, Sioux Falls, SD 57104 USA; 4 Boekelheide Neonatal Intensive Care Unit, Sanford Children’s Hospital, 1600 W. 22nd St., PO Box 5039, Sioux Falls, SD USA

**Keywords:** Breast milk fatty acids, Docosahexaenoic acid, Omega-3 fatty acids, Long chain polyunsaturated fatty acids, Breastfeeding

## Abstract

**Background:**

Docosahexaenoic acid (DHA), an omega-3 fatty acid found in breast milk, has many health benefits for both mother and baby. A 2007 meta-analysis found U.S. women had breast milk DHA levels (0.20% of total fatty acids) below the worldwide mean (0.32%). In 2008, international dietary recommendations were made for pregnant and lactating women to consume 200 mg of DHA per day. This community-based study aimed to define current milk DHA levels from upper Midwest USA lactating mothers and to determine if providing information about their own level along with dietary recommendations would incite changes to increase breast milk DHA content.

**Methods:**

New mothers attending lactation classes or using hospital pumping rooms in Sioux Falls, South Dakota, USA participated by providing one drop of breast milk on a card for fatty acid analysis at baseline and 1 month after initial reporting. DHA levels were analyzed by gas chromatography. Mothers received a report of their own breast milk level along with dietary recommendations on DHA intake for lactating women. Median baseline and follow-up DHA levels were determined and differences were compared by Wilcoxon signed-rank test.

**Results:**

At baseline, breast milk DHA content (*n* = 84) was highly variable (range 0.05 to 0.73%) with a median of 0.18% (IQR, 0.13, 0.28; mean ± SD, 0.22 ± 0.13%), well below the worldwide average (0.32%). Women who reported taking DHA supplements (*n* = 43) had higher levels than those who did not (0.23% vs. 0.15%, *P* < 0.0001). In a subset of 60 mothers who submitted a second sample, median breast milk DHA content increased from 0.19 to 0.22% (*P* < 0.01).

**Conclusions:**

Findings suggest that providing nursing mothers with their breast milk DHA level and education about DHA intake while breastfeeding motivates change to increase DHA levels.

**Electronic supplementary material:**

The online version of this article (doi:10.1186/s13006-016-0099-0) contains supplementary material, which is available to authorized users.

## Background

Long-chain polyunsaturated fatty acids (LCPUFAs) are essential nutrients required for normal health, growth and development. Of these, arachidonic acid (ARA, an omega-6 fatty acid) and docosahexaenoic acid (DHA, an omega-3 fatty acid) are especially important for the health of both mother and the developing infant who relies on provision from breast milk or formula. Mother’s milk is the best nutrition for babies in the first 6 months of life and it contains all the essential LCPUFAs. Although breast milk levels of ARA are fairly stable, breast milk levels of DHA are largely determined by maternal blood levels and influenced by intake, whether from fish (which can vary markedly around the world) or DHA supplementation (e.g., omega-3 or fish oil supplements) [1, 2]. DHA has important health benefits for pregnant and lactating mothers and increasing evidence shows that infants fed breast milk with a higher DHA content have better vision and neurodevelopmental outcomes [3–9].

A meta-analysis based on studies published between 1986 and 2006 reported that the estimated worldwide average (WWA) level of DHA in human milk was a mean of 0.32 ± 0.22% with a wide range of 0.06 to 1.4% of total fatty acids [10]. Since 2007, this value has been adopted by many infant formula companies as a target for DHA content in their products. The assumption that the WWA should be considered the target has been both questioned [11] and supported [4]. On average women in the US have breast milk DHA levels below this reported WWA. Based on ten studies completed before 2007 (mean n per study = 23), the average breast milk DHA level in US mothers was 0.19% (Table 1). In seven US studies published after 2007 (six studies with mean *n* = 38 and one study with a sample *n* = 287), the average breast milk DHA level was still below the WWA at 0.17%.

**Table 1:** Overview of studies of breast milk DHA before and after a 2007 review [10]

Reference	Site	Participants	DHA (% wt)
Studies published before 2007			
Harris et al., 1984 [47]	Oregon, US	8	0.10%
Carlson et al., 1986 [48]	US	11	0.19%
Henderson et al., 1992 [29]	Connecticut, US	5	0.37%
Francois et al., 1998 [49]	Oregon, US	14	0.20%
Jensen et al., 2000 [1]	US	24	0.24%
Auestad et al., 2001 [50]	US	43	0.12%
Francois et al., 2003 [37]	Oregon, US	7	0.20%
Bopp et al., 2005 [51]	North Carolina, US	22	0.21%
Jensen et al., 2005 [6]	Texas, US	77	0.20%
Yuhas et al., 2006 [52]	US	49	0.17%
Average ^a^		260	0.19%
Studies published after 2007			
Glew et al., 2008 [53]	New Mexico, US	29	0.11%
Valentine et al., 2010 [54]	Ohio, US	39	0.10%
Glew et al., 2011 [55]	New Mexico, US (American Indian)	19	0.10%
Baack et al., 2012 [56]	Iowa, US	31	0.07%
	Texas, US	5	0.20%
	North Carolina, US	5	0.15%
	California, US	5	0.14%
Keim et al., 2012 [57]	North Carolina, US	287	0.20%
Valentine et al., 2013 [58]	Ohio, US	13	0.18%
Sherry et al., 2015 [36]	US	82	0.18%
Average ^a^		515	0.17%

^a^Weighted mean by study *n*

The apparent lack of change in breast milk DHA levels in the US may be due to conflicting advice regarding recommended DHA intake and the safety of fish consumption during pregnancy and lactation. In the early 2000s, recommendations were made by the US Food and Drug Administration that pregnant and lactating women should limit their intake of fish that could contain high levels of methyl-mercury [12], which was followed by a decline in fish consumption by pregnant women [13]. In 2008, international recommendations were made for pregnant and lactating women to consume 200 mg of DHA per day, ideally from low mercury fish [14]. These seemingly conflicting recommendations may have inadvertently led to a decrease in DHA intake in lactating women. This is evidenced by the observation that breast milk DHA levels have not increased since the 2008 recommendation.

There is an ongoing need for up-to-date information on what “normal” human milk DHA levels are for American women and how women can safely increase them. Accordingly, the objective of this study was to determine what current breast milk DHA levels are in lactating women in the upper Midwest USA (a region not previously examined). The second goal of this study was to explore whether informing mothers of their own milk’s DHA along with dietary recommendations would incite changes to increase breast milk DHA.

## Methods

### Recruitment

Participants were recruited from breastfeeding classes, community breastfeeding support groups and pumping rooms at Sanford Health in Sioux Falls, South Dakota, USA via displayed materials and available study packets that explained the eligibility criteria. Study packets included an invitation letter, a consent form, two sample collection cards (baseline and 1 month with instructions for use), two brief questionnaires (baseline and 1 month), and two pre-paid, return mail envelopes (baseline and 1 month). Women over 18 years of age, lactating for at least 1 week, planning to breastfeed for at least an additional 4 weeks, and able to read English were included in the study. Women who met the inclusion criteria were enrolled when they voluntarily returned a signed informed consent, a dried mother’s milk sample, and a completed baseline questionnaire.

### Fatty acid analysis

One drop of breast milk was placed on a filter paper collection card [15] and mailed to the laboratory (OmegaQuant Analytics, LLC; Sioux Falls, SD) for measurement of fatty acid content. Fatty acids were determined using gas chromatography and levels are presented as a percent of total fatty acids as previously described and validated [16]. In short, dried breast milk samples were combined with the methylating mixture [boron trifluoride in methanol (14%), toluene and methanol (35/30/35 v/v)], shaken and heated at 100 °C for 45 min. After cooling, equal volumes of hexane and distilled water were added. Samples were briefly vortexed, spun to separate layers, and an aliquot of the hexane (upper) layer which contained the fatty acid methyl esters was taken for analysis by gas chromatography as described previously [17].

### Feedback and follow-up

Participants received their baseline breast milk DHA level and dietary recommendations via an emailed report within 1 week of sending in their sample. The report gave different instructions to the mother depending on her DHA level. If her level was below the WWA (0.32% DHA), she was advised to increase her DHA intake via low-mercury containing fish and/or a DHA supplement (with a target of 200 mg DHA/day as recommended [14]). To assist with this, a table showing the DHA and mercury content of a variety of seafood and supplements was included with the report (Additional file 1; Fish intake). If her breast milk DHA level was at or above the WWA, she was encouraged to maintain her current level of DHA intake. A second breast milk sample was collected and returned with another brief questionnaire 4 weeks after the first sample was taken.

### Survey collection

Although simple, the questionnaires were designed to determine what changes, if any, had been made to fish intake and/or the use of DHA supplements in response to the information provided with the first DHA value (Additional files 2 and 3). One-page surveys were voluntarily filled out and returned by participants in pre-paid, return mail envelopes. The baseline survey collected information about demographic and pregnancy characteristics, average servings of fish per month and the use of a DHA containing supplement (Additional file 2; Baseline questionnaire). The follow-up survey simply asked if the first mother’s milk DHA test motivated change in diet or supplementation (Additional file 3; Follow-up questionnaire).

### Statistical analysis

Data are presented as medians (Interquartile Range [IQR]; 25^th^ percentile, 75^th^ percentile) due to the non-normal distribution for most variables. Categorical variables, i.e. race/ethnicity in all participants versus those participants who provided a follow-up sample, were compared using Fisher’s Exact test. Mann-Whitney test was used to compare baseline continuous variables, including DHA levels, between independent groups. Wilcoxon signed-rank test was used to compare the difference between baseline and follow-up DHA levels in participants who provided a follow-up sample after being informed of their initial DHA level. Significance was set at *P* < 0.05. Data were analyzed using GraphPad Prism 6 (GraphPad software, San Diego, CA, US).

## Results

**Fig. 1:**
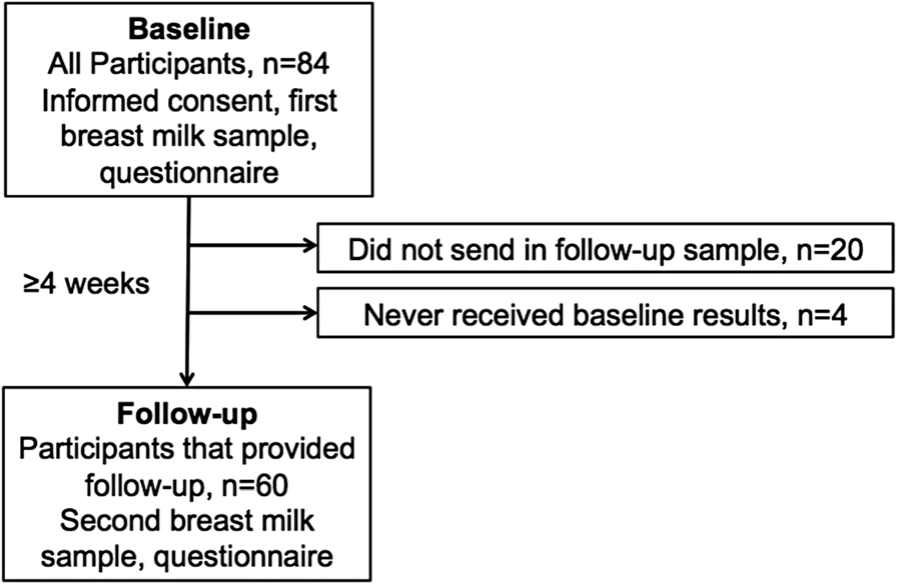
Diagram of study design and participant flow

Baseline data were available from 84 women. Of them, 20 did not return their follow-up sample and questionnaire (Fig. 1). For six women, email difficulties prevented them from receiving their baseline breast milk DHA results (apparently due to spam email filters). Four of these six women still sent in their follow-up sample despite not receiving their baseline report, but this data was not used in the main analyses as they were unable to make an informed decision about whether to change their DHA intakes without baseline information. The reasons why the other 20 mothers (including two who did not receive their baseline results) did not send follow up samples are unknown. Therefore, 60 women were included in the follow-up analyses.

Participants who provided a follow-up sample included 71% of all participants and there was no statistically significant differences in baseline characteristics between women who provided follow-up sample and those who did not (Table 2). On average, the women who provided baseline breast milk samples were 30 years old, had a pre-pregnancy BMI of 24 kg/m^2^, had two children, were white/Caucasian, and were relatively highly educated. There were no statistically significant baseline differences between the cohort of women who provided the follow-up data (*n* = 60) and those not included in the follow-up analysis (*n* = 24); however, there was weak evidence for a difference in pre-pregnancy BMI (*P* = 0.055).

**Table 2:** Baseline characteristics and DHA levels of study participants

Variable ^a^	All participants	Participants who provided a follow-up sample
*N*	84	60
Age (years)	30 (27, 32)	30 (27, 32)
Pre-pregnancy BMI (kg/m^2^)	24.2 (21.7, 28.1)	23.6 (21.1, 26.6)
Race/Ethnicity (% White/Caucasian)	99% (83)	98% (59)
Education (Associates Degree or more)	87% (73)	85% (51)
Parity (number of pregnancies)	2 (1, 3)	2 (1, 3)
Gestation length (weeks)	39 (38, 40)	39 (38, 40)
Lactation length (weeks)	15.3 (6.0, 28.8)	12.0 (6.0, 22.8)
Pregnancy weight gain (kg)	15.9 (13.6, 22.7)	15.9 (13.6, 23.8)
Percent of mothers who had diabetes during pregnancy (n)	7% (6)	7% (4)
Percent of mothers supplementing with DHA during lactation (baseline; n)	51% (43)	53% (32)
Percent of mothers who supplemented with DHA during pregnancy (n)	72% (59)	71% (42)
Breast milk DHA level (% of total fatty acids)	0.18% (0.13, 0.28)	0.19% (0.15, 0.30)
Percent of mothers with milk DHA ≥ 0.32% (Worldwide Average ^b^; n)	17% (14)	18% (11)

*DHA* docosahexaenoic acid, *BMI* body mass index

^a^All variables except for breast milk DHA levels were from self-reported data collected at baseline. Medians and interquartile ranges (IQR) presented and compared using Mann-Whitney (continuous) and Fisher’s Exact (categorical) statistical tests. No differences between groups were detected

^b^Worldwide average as estimated by Brenna et al. [10]

**Fig. 2:**
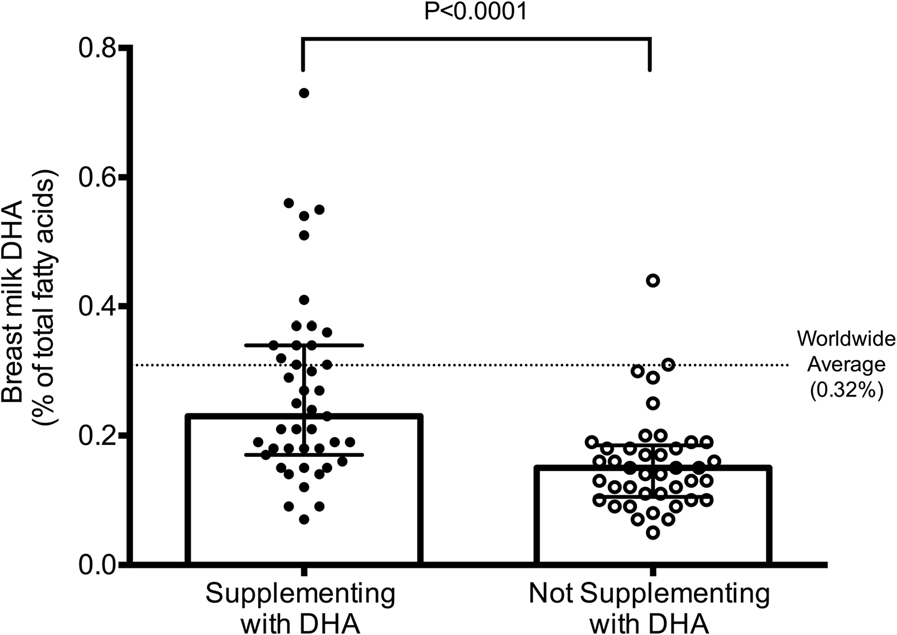
Baseline mother’s milk DHA levels. Comparison of baseline milk DHA levels between women who reported taking DHA supplements (0.23%, IQR 0.17, 0.34; *n* = 43) and those who did not (0.15%, IQR 0.11, 0.19; *n* = 41) during lactation. Group medians compared with Mann-Whitney statistical test. Symbols (*dots*) represent individual participant data, bar represents median, lines represent IQR. Worldwide average breast milk DHA level from Brenna et al [10] is indicated by the dashed line

Mother’s milk fatty acid profiles are reported in Table 3. Baseline breast milk DHA levels from mothers in our study (from the upper Midwest USA) were highly variable (range 0.05 to 0.73%) with a median value of 0.18% (IQR: 0.13, 0.29; mean ± SD, 0.22 ± 0.13%) which is below the WWA (0.32%), but consistent with previous studies in North American women (see Table 1). The referenced WWA^10^ included data from non-supplementing women; to understand how non-supplementing women in the upper Midwest USA compare, the average mother’s milk DHA level of the non-supplementing women in our baseline cohort (*n* = 43/84, 51%) was also calculated. The median mother’s milk DHA level for non-supplementing women was 0.15% (IQR: 0.11, 0.19) which is significantly lower than the WWA and also significantly lower than those women in our study who reported taking a supplement (IQR: 0.15% vs. 0.23%, *P* < 0.0001; Fig. 2). Women who reported taking a DHA supplement during their last trimester of pregnancy (*n* = 59/84, 70%) also had higher breast milk DHA levels than those who did not report this (0.19% vs. 0.14%, *P* < 0.01). Only 14 women in our study had baseline breast milk DHA levels greater than the WWA. Of them, 13 reported taking a DHA supplement. Despite this, 42% (*n* = 30/70) of women whose baseline breast milk DHA level was below the WWA reported taking a DHA-containing supplement. This suggests that the routine supplementation reported was not adequate to raise mother’s milk levels to the WWA.

**Table 3:** Fatty acid profile of breast milk in baseline (*n* = 84) and follow-up cohorts (*n* = 60)

Fatty Acids	All Participants (*n* = 84) ^1^	Participants who provided a follow-up sample (*n* = 60)^1^		*P*-value ^2^
		*Baseline*	*4 weeks*	
N6:N3	12.10 (10.28, 13.88)	11.65 (9.70, 13.85)	11.10 (9.43, 12.65)	0.10
AA:EPA	9.61 (5.82, 12.66)	9.57 (5.52, 13.95)	6.63 (3.52, 10.74)	0.0008*
SAT	33.35% (29.73, 35.68)	33.50% (30.08, 35.60)	33.50% (31.13, 37.03)	0.06
MUFA	36.65% (34.13, 39.93)	37.25% (35.63,41.78)	36.80% (34.00, 39.90)	0.32
N6	18.80% (16.33, 21.88)	18.65% (16.75, 22.48)	18.60% (15.30, 21.75)	0.11
N3	1.60% (1.30, 1.98)	1.60% (1.33, 2.18)	1.60% (1.40, 2.10)	0.91
TRANS	1.70% (1.30, 2.20)	1.65% (1.20, 2.20)	1.75% (1.40, 2.28)	0.42
C10:0	1.00% (0.80, 1.38)	1.10% (0.90, 1.48)	1.10% (0.90, 1.30)	0.51
C12:0	5.10% (3.43, 6.60)	5.50% (3.43, 6.90)	5.30% (4.00, 6.98)	0.05
C14:0	5.65% (4.20, 6.98)	6.00% (4.25, 7.55)	5.90% (4.40, 7.30)	0.03
C16:0	20.55% (19.20, 22.18)	20.10% (19.03, 22.43)	20.20% (18.88, 22.45)	0.49
C16:1n7t	0.10% (0.10, 0.10)	0.10% (0.10, 0.10)	0.10% (0.10, 0.10)	>0.99
C16:1n7	1.75% (1.30, 2.38)	1.85% (1.40, 2.50)	1.80% (1.43, 2.10)	0.97
C18:0	6.35% (5.43, 7.20)	6.35% (5.50, 7.10)	6.80% (5.90, 7.88)	0.04
C18:1 t	1.20% (0.90, 1.70)	1.20% (0.90, 1.70)	1.30% (1.00, 1.70)	0.41
C18:1n9	34.75% (32.25, 38.08)	34.90% (32.70, 39.78)	34.25% (31.78, 37.13)	0.29
C18:2n6tt	0.40% (0.30, 0.40)	0.30% (0.30, 0.40)	0.30% (0.30, 0.40)	0.70
C18:2n6	17.45% (15.30, 20.83)	16.90% (15.30, 20.98)	17.30% (14.10, 20.03)	0.13
C20:0	0.20% (0.20, 0.20)	0.20% (0.20, 0.20)	0.20% (0.20, 0.20)	0.57
C18:3n6	0.20% (0.10, 0.20)	0.20% (0.10, 0.20)	0.20% (0.10, 0.20)	0.13
C20:1n9	0.30% (0.30, 0.40)	0.30% (0.30, 0.40)	0.30% (0.30, 0.40)	0.81
C18:3n3	1.20% (0.90, 1.50)	1.20% (0.90, 1.68)	1.10% (1.00, 1.40)	0.47
C20:2n6	0.30% (0.30, 0.40)	0.30% (0.30, 0.40)	0.30% (0.20, 0.30)	0.003*
C22:0	0.10% (0.10, 0.10)	0.10% (0.10, 0.10)	0.10% (0.10, 0.10)	0.29
C20:3n6	0.35% (0.30, 0.40)	0.40% (0.30, 0.50)	0.30% (0.30, 0.40)	<0.0001*
C20:4n6	0.45% (0.40, 0.50)	0.50% (0.40, 0.50)	0.40% (0.40, 0.50)	0.01*
C24:0	0.10% (0.00, 0.10)	0.10% (0.00, 0.10)	0.10% (0.00, 0.10)	0.73
C20:5n3	0.00% (0.00, 0.10)	0.00% (0.00, 0.10)	0.10% (0.10, 0.10)	0.004*
C24:1n9	0.00% (0.00, 0.00)	0.00% (0.00, 0.00)	0.00% (0.00, 0.00)	0.48
C22:4n6	0.10% (0.10, 0.10)	0.10% (0.10, 0.10)	0.10% (0.10, 0.10)	0.09
C22:5n6	0.00% (0.00, 0.00)	0.00% (0.00, 0.00)	0.00% (0.00, 0.00)	>0.99
C22:5n3	0.10% (0.10, 0.20)	0.10% (0.10, 0.20)	0.10% (0.10, 0.20)	0.19
C22:6n3	0.18% (0.13, 0.29)	0.19% (0.14, 0.30)	0.22% (0.16, 0.35)	0.007*

*Abbreviations*: *N6:N3* omega-6:omega-3 fatty acid ratio, *AA:EPA* arachidonic acid: eicosapentaenoic acid ratio, *SAT* total saturated fatty acids, *MUFA* total monounsaturated fatty acids, *N6* total omega-6 fatty acids, *N-3* total omega-3 fatty acids, *TRANS* total *trans* fatty acids

^1^ Medians (IQR)

^2^ Significance was set at *P* ≤ 0.01* (due to multiple variables) comparing the baseline and follow-up samples from participants who provided a follow-up sample

In our study, the reported intake of fish did not correlate with mother’s milk DHA levels: “Less than 1 serving/month” 0.18% (IQR: 0.14, 0.24; *n* = 24); “1 to 3 servings/month” 0.18% (IQR: 0.11, 0.31; *n* = 47); “4 or more servings/month” 0.18% (IQR: 0.15, 0.30; *n* = 13).

**Fig. 3:**
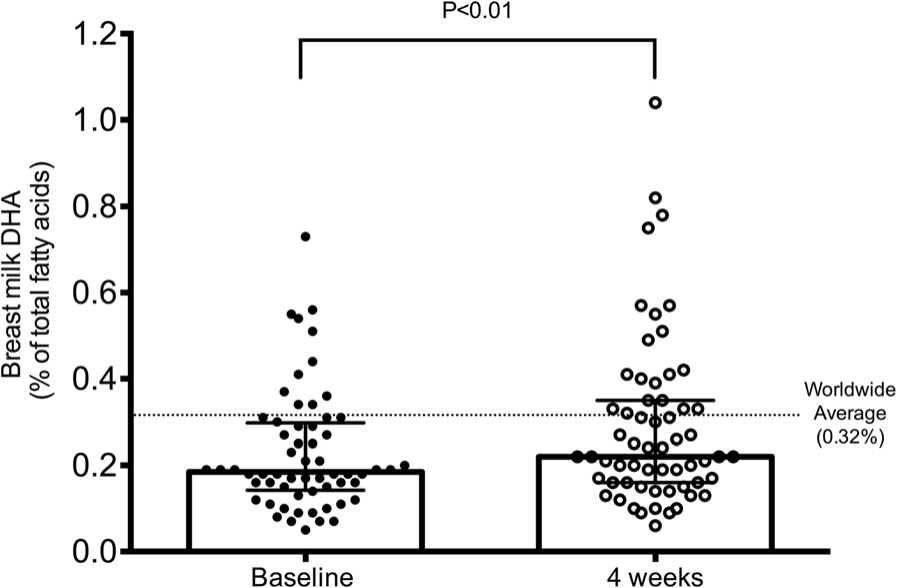
Mother’s Milk DHA Levels from Participants who Provided Follow-up. Milk DHA levels at baseline (0.19%, IQR, 0.14, 0.30) and at least 4 weeks later (0.22%, IQR, 0.16, 0.35) from participants who provided follow-up (*n* = 60). Group medians compared with the Wilcoxon statistical test. Symbols (*dots*) represent individual participant data, bar represents median, lines represent IQR. Worldwide average breast milk DHA level from Brenna et al. [10] indicated by a *dashed line*

Follow-up samples (*n* = 60) were collected 38 ± 17 days after the baseline sample. Median mother’s milk DHA levels increased significantly from baseline in this cohort (0.19 to 0.22%, *P* < 0.01; Fig. 3). Whether women had baseline breast milk DHA levels higher (*n* = 11) or lower (*n* = 49) than the WWA did not affect the magnitude of the change in DHA levels (−0.03% vs. +0.04%, respectively; *P* = 0.40. Forty-three of 60 women in the follow-up cohort responded to questions about the extent to which receiving information on their breast milk DHA levels motivated them to make dietary and/or supplementation changes. Of them, 51% (*n* = 22/43) reported changing their diet and 57% (*n* = 25/43) reported changing their supplementation habits.

## Discussion

There were two primary findings of this study. First, mother’s milk DHA levels in the Midwestern community of Sioux Falls, SD, USA averaged about 0.22%, which correlates with other North American studies, but is well below the WWA of 0.32%. This finding is disconcerting given 2007 guidelines to increase DHA provision during pregnancy and lactation for the health of both mother and her baby [14]. Although most women in our cohort reported eating fish, mother’s milk DHA levels did not correlate with fish intake. Reporting the use of a DHA supplement did correlate with mother’s milk DHA levels in our study. In this upper Midwestern USA cohort, 46% of the women reported taking a DHA supplement, and yet their average breast milk DHA level was still one-third lower than the WWA. Women who reported not taking a DHA supplement during lactation had even lower breast milk DHA levels, at about 50% of the WWA. Although compliance was not monitored in this study, findings suggest that routine guidelines for DHA supplementation during lactation may not be enough to increase mother’s milk DHA levels to the WWA.

The second finding of this study is that when women were provided with information about their breast milk DHA levels and information on the recommended intake for lactating mothers, they responded with a modest increase in milk DHA content within about 1 month. This suggests that mother’s milk DHA testing, alongside written educational information, may be an efficient and cost effective approach to motivate lactating women to improve their DHA status and in turn, that of their nursing infant.

DHA, an omega-3 LCPUFA, is an essential nutrient important for both maternal and infant health [5, 18]. For the pregnant woman, DHA status is directly associated with the length of gestation [19] and fetal growth [20] and is indirectly associated with anxiety [21] and postpartum depressive symptoms [5]. Indeed, supplementation during pregnancy may even decrease healthcare costs [22]. The developing baby receives LCPUFAs primarily from a maternal source and mother’s blood and milk DHA levels directly correlate with DHA levels in infants [2]. For the baby, DHA supports vision, brain development, memory [23], attention [24] and immunological status [25]. Indeed, improved DHA status during infancy may have lasting effects on learning [26] and is associated with less atopic disease [5, 25].

Human milk contains all essential LCPUFAs, but unfortunately, DHA levels are extremely variable and dependent on maternal intake [9, 10]. There are very few outcomes based studies conducted in infants who are exclusively breastfed with varying levels of DHA when compared to studies conducted in infants who are fed formula with varying DHA content. Thus, the optimal breast milk DHA level has yet to be determined. There is evidence to suggest that breast milk DHA levels of 0.32% or greater confers added benefit for the infant’s neurodevelopment as compared to lower DHA values (~0.2%), which are typical of US mothers [2, 4, 6, 27]. Unfortunately, in the WWA report [10], the coefficient of variability across populations for DHA was 69% whereas that for ARA was only 28%. This variability in DHA levels is based on many factors, including maternal age [28], genetic variation, race, gestation, body mass index, parity, smoking, duration of lactation, and most importantly, maternal diet [1, 29–35]. Maternal consumption of an appropriate ratio of ω-3 and ω-6 fatty acids and adequate preformed DHA intake directly correlate with breast milk DHA levels [9, 36]. Increasing intake of alpha-linolenic acid, the plant-based precursor to DHA, does not significantly increase breast milk DHA levels [37]. Therefore, a minimum daily DHA intake of 200 mg/day is recommended for pregnant and lactating women to support both maternal and infant health [14, 38].

It has been clearly shown that supplementation increases mother’s milk DHA content [25, 33, 39]. Unfortunately, in our study women who report taking a DHA supplement still had breast milk DHA levels below the WWA. Although compliance and average daily DHA doses were asked for from our study participants, these specific response rates were low making it difficult to draw any insight about optimal supplementation doses. Similarly, formal dietary assessment was not conducted in this study, so background dietary intake of DHA cannot be ascertained. According to the 2009–2010 National Health And Nutrition Survey (NHANES), women 20–39 years of age have an average DHA intake only 50–60 mg/day [40]. By comparison, DHA intake in pregnant women in Japan is over 300 mg/day [41] and the three Japanese studies reported in the meta-analysis by Brenna et al. [10] had breast milk DHA levels of 0.53, 0.99 and 1.1%. Even women in parts of the world where fish intake is not as high as it is in Japan have higher mother’s milk DHA levels than the women in our study [33, 42, 43]. Moreover, others have found that using a higher daily dose of DHA during pregnancy and lactation is safe (≥800 mg/day [44]), increases both maternal and infant levels and may provide additional health benefits [9].

Although mother’s milk DHA levels in our study were lower than the WWA, we observed that a majority of women who were informed about their breast milk DHA level and given information about how to safely increase it were motivated to do so. This was evidenced by their responses to questions about changes made to diets or supplementation habits and by a significant increase in mother’s milk DHA levels 5–6 weeks later. Since DHA levels in mature milk typically remain the same or decline over time [30, 33, 34], we believe the observed increase was due to increased nutritional DHA intake (primarily from supplements) and not to other biological factors. Other influences associated with maternal DHA status such as race, gestation, and body weight [33, 45, 46] did not change within our study population. Because increased DHA status is associated with positive maternal and neonatal outcomes, and the guidelines alone have not changed mother’s milk DHA levels, we recommend that breastfeeding mothers should be routinely counseled about the benefits of supplementing DHA during lactation [14]. We also concur that testing and informing mothers about their breast milk DHA levels could be a novel and cost-effective way to improve DHA status and would provide measurable values necessary to guide ongoing adjustment in the recommended daily dose of DHA during pregnancy and lactation to optimize outcomes for both mother and baby.

## Limitations and advantages

A limitation of this study was that the cohort was racially homogeneous and relatively well-educated, which undoubtedly introduced some selection bias. Regardless, mother’s milk DHA levels still were generally low. All mothers in this study lived in the upper Midwest of the USA and in a single community (a review of past studies [Table 1] suggests that single city studies are the norm in this research arena). Since there is little data in the literature on milk FA status from women in this region, this study fills a gap in knowledge and helps us understand the community’s response to routine recommendations and additional counseling of lactating mothers. Another limitation is that follow up was not complete in all participants. Several mothers did not return a second test within a month of getting the report on their breast milk DHA levels. Accordingly, we cannot properly determine the extent to which providing this education motivated change since the group of women who did send a second sample may not be representative of all women in the study. However, baseline characteristics of those who did and did not submit a follow up sample were similar.

An additional study limitation was our dependence on self-report for demographic, dietary and supplement use information. The questionnaire was simple and did not include a validated food diary or the timing of sample collection during the feed (before, during or after a breastfeed). The use of a validated food diary would provide valuable information about maternal diet, but may have hindered participation and compliance. While timing of sample collection affects the measured total fat content of milk, it should not affect the percent composition of fatty acids in mother’s milk which was used in this study. Possible misunderstandings of certain items in the baseline questionnaire (e.g., parity, duration of lactation, etc.) and failing to complete the entire survey could have been avoided with a real-time interview; however, this type of interaction may have introduced persuasion bias. Additionally, there was no control group that did not receive feedback on their breast milk DHA levels, and so we cannot strictly attribute the changes in dietary/supplementation behavior and breast milk DHA levels to the feedback and education provided.

Strengths of the study include that it is a relatively large sample size (for this type of project) being the second largest of the 17 studies included in Table 1. This is the only study to our knowledge that explores the effects of informing the subjects of their own personal levels on breast milk DHA levels. We also used a novel and validated dried milk spot method to collect and measure milk DHA content. The test is simple to use, with little to no risk and is commercially available; whether the cost outweighs the benefit remains to be seen.

## Conclusions

Despite current guidelines aimed to support DHA status during pregnancy and lactation, breastfeeding mothers in this upper Midwestern USA city have mother’s milk DHA levels that are lower than the WWA, even with reported fish or supplement intake. However, breast milk DHA content can be increased with personalized testing and the provision of information about safe ways to attain the recommended daily DHA intake during lactation. Offering mother’s milk DHA testing and further education about the importance of DHA during lactation may advance the understanding of optimal provision for breastfeeding mothers and improve health outcomes for mothers and their babies.

## Additional files

Additional file 1: Fish intake. (JPG 235 kb)
Additional file 2: Baseline questionnaire. (PDF 83 kb)
Additional file 3: Follow-up questionnaire. (PDF 331 kb)

## Abbreviations

ARA: Arachidonic acid
BMI: Body mass index
DHA: Docosahexaenoic acid
IQR: Interquartile range
LCPUFA: Long chain polyunsaturated fatty acid
WWA: World-wide average
