# Supplementary figures and images for: Breast milk DHA levels may increase after informing women: a community-based cohort study from South Dakota USA
Source: Int Breastfeed J. 2017 Jan 28;12:7. doi: 10.1186/s13006-016-0099-0 (PMC5273852; doi:10.1186/s13006-016-0099-0)

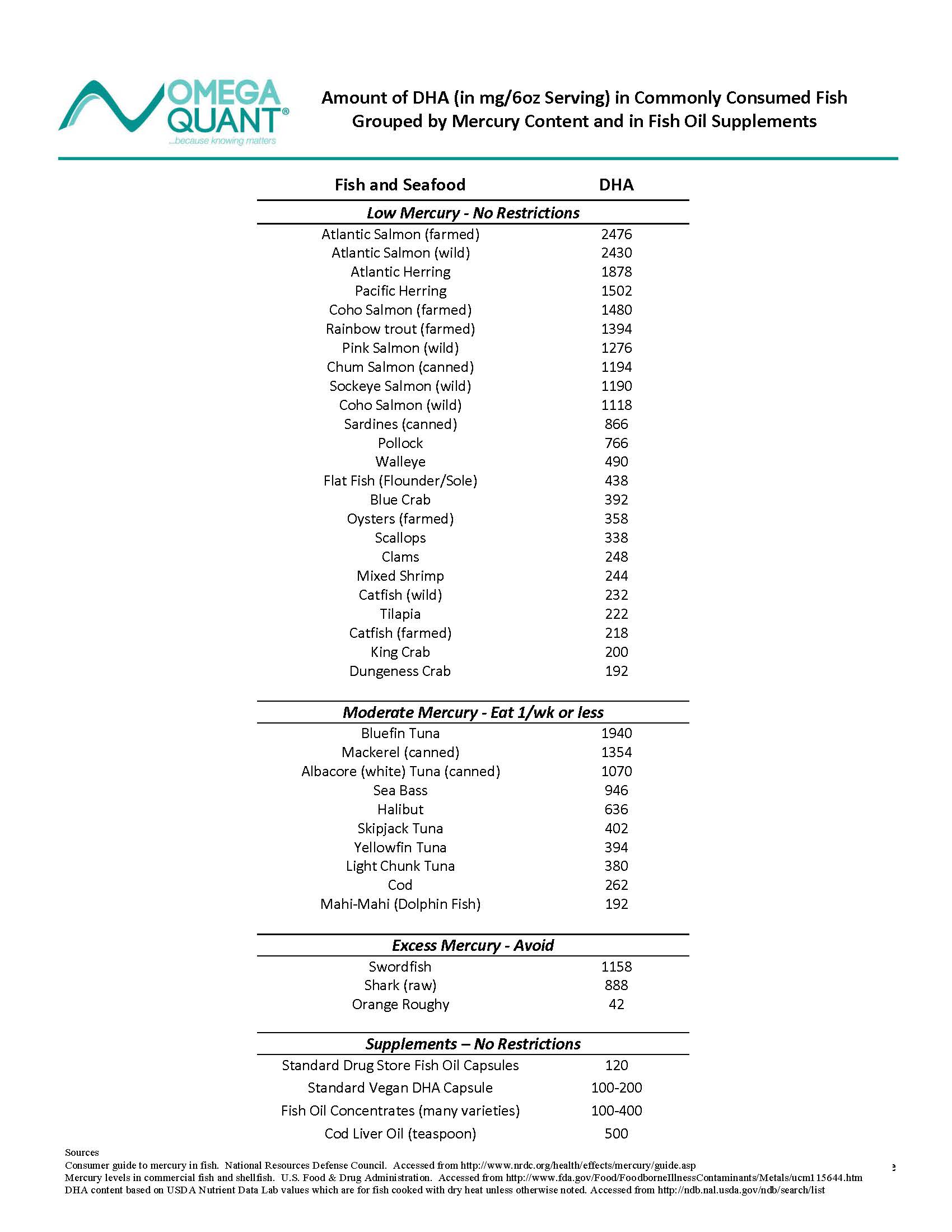

Supplement: Additional file 1: — Fish intake. (JPG 235 kb) [file 13006_2016_99_MOESM1_ESM.jpg]
