# Supplementary material for: Breast milk DHA levels may increase after informing women: a community-based cohort study from South Dakota USA
Source: Int Breastfeed J. 2017 Jan 28;12:7. doi: 10.1186/s13006-016-0099-0 (PMC5273852; doi:10.1186/s13006-016-0099-0)
Supplement: Additional file 2: — Baseline questionnaire. (PDF 83 kb) [file 13006_2016_99_MOESM2_ESM.pdf]

Subject No. _____ (to be completed by research staff)

Baseline Questionnaire

Mother's Milk DHA Study
University of South Dakota School of Medicine

Please fill out this short questionnaire and send it in with your first dried milk sample. If you have any questions, please do not hesitate to contact us at info@omegaquant.com. Thank you for your participation!

1. **Name** _________________________________ 2. **Email** ________________________________

3. **Home Address** ____

4. **Phone** _________________________________

5. **Age** _______

6. **Race/ethnicity (optional; circle)** Asian/Pacific Islander Black/African American Hispanic American Indian White/Caucasian

7. **Pre-Pregnancy Height** _______ft _____ in

8. **Pre-Pregnancy Weight** ___________ pounds

9. **Highest Education** Less than high school High school Some college Associate's degree Bachelor's degree Post-Graduate Degree (i.e., Masters, Doctoral, etc.)

10. **Number of total pregnancies** ____________

11. **Do you currently take a DHA supplement (fish oil, krill oil, or prenatal vitamin)**?
Yes No
If **yes**, please indicate Number of pills per week ____________
Amount of DHA per pill ____________ mg*

12. **During your last trimester, did you regularly take prenatal vitamin containing DHA**?
Yes No If **yes**, please indicate the amount of DHA per pill _____ mg*

13. **How many times per month on average do you eat tuna or other non-fried fish as a main course**? _________

14. **How much weight did you gain during this past pregnancy**? _____pounds

15. **At how many weeks gestation did you deliver**? ___ weeks

16. **How long have you been lactating?** ____ weeks

17. **Did you have diabetes during this past pregnancy**? Yes No

18. **At what time of day did you collect (are you collecting) the milk sample?** ____ AM / PM

- if you don't remember these details, you can answer them on the second questionnaire which will be sent to your home
