# Supplementary material for: Breast milk DHA levels may increase after informing women: a community-based cohort study from South Dakota USA
Source: Int Breastfeed J. 2017 Jan 28;12:7. doi: 10.1186/s13006-016-0099-0 (PMC5273852; doi:10.1186/s13006-016-0099-0)
Supplement: Additional file 3: — Follow-up questionnaire. (PDF 331 kb) [file 13006_2016_99_MOESM3_ESM.pdf]

Mother's Milk DHA Study - Follow-up Questionnaire

Please fill out this short follow-up questionnaire and return it along with your dried milk sample. If you have any questions, please do not hesitate to contact any of the investigators conducting this study at info@omegaquant.com. Thank you for your participation!

Bill Harris, PhD
Michelle Baack, MD
Brian Juber, MS-3, Sanford School of Medicine

1. **Name**: ________________________________

2. **Please indicate <u>any changes</u> in your contact information**: ___________________________________

____

3. **Did the results of your first Mother's Milk DHA Test motivate you to <u>change your diet</u>**? Yes No

If yes, please indicate which food(s) you used to increase your intake of DHA:

_______ Fish
- _______ Salmon
- _______ Tuna
- _______ Herring
- _______ Trout
- _______ Sardines
- _______ Crab
- _______ Oysters
- _______ Clams
- _______ Shrimp
- _______ Catfish
- _______ Tilapia
- _______ Other (please indicate below)

_______ Other foods rich in omega-3's
- _______ Flaxseed
- _______ Walnuts
- _______ Soybeans
- _______ Tofu
- _______ Brussels sprouts
- _______ Cauliflower
- _______ Squash
- _______ Other (please indicate below)

____

How many days per week did you eat the above food? ____________________________

4. **Did the results of your first Mother's Milk DHA Test motivate you to <u>start taking a supplement</u>**?
Yes No
If yes, how many pills per week did you take? _________ pills
If yes, please indicate the amount of DHA contained in each capsule: _____________ mg

Please complete and return this form with your repeat milk spot sample to OmegaQuant® in the envelope provided.
*Submission of this milk sample for analysis indicates that the person named above understands the purpose and procedures of this study and thereby agrees to participate.
